# Incidence and predictors of chronic kidney disease among hypertensive patients in Ethiopia: A Bayesian multivariate joint model

**DOI:** 10.1371/journal.pone.0334428

**Published:** 2025-10-10

**Authors:** Dejen Kahsay Asgedom, Habtamu Wagnew Abuhay, Lemma Derseh

**Affiliations:** 1 Department of Public Health, College of Medicine and Health Sciences, Samara University, Samara, Ethiopia; 2 Department of Epidemiology and Biostatistics, Institute of Public Health, College of Medicine and Health Sciences, University of Gondar, Gondar, Ethiopia; Universita degli Studi Magna Graecia di Catanzaro, ITALY

## Abstract

**Background:**

Hypertension (HTN) is a major contributor to chronic kidney disease (CKD), a leading cause of global morbidity and mortality. In low-resource settings such as Ethiopia, where CKD risk factors remain understudied, identifying predictors and longitudinal blood pressure (BP) patterns associated with CKD incidence is crucial for early intervention. Therefore, this study aimed to determine the incidence and predictors of CKD, as well as its association with longitudinal BP changes, among hypertensive patients in Ethiopia.

**Methods:**

An institution-based retrospective follow-up study was conducted at the University of Gondar Comprehensive Specialized Referral Hospital. Using a Bayesian joint modeling approach, we integrated Cox proportional hazard and linear mixed effects models to evaluate the effects of time-dependent BP trajectories on CKD risk. The data were entered into the Kobo toolbox and analyzed with R software (version 4.3.1).

**Results:**

A total of 408 hypertensive patients were followed for 2322.83 person-years. At the end of the follow-up, 58/408 (14.22%) developed CKD, with an incidence density of 2.5 cases per 100 person‐years (95% CI: 1.89–3.14). Both the current values and longitudinal the quarterly rate of change in BP were significantly associated with increased CKD risk. For systolic BP, the adjusted hazard ratio (AHR) was 6.25 (95% CrI: 2.85–9.85) for the current values and 3.75 (95% CrI: 3.16–7.95) for the quarterly rate of change. Similarly, the diastolic BP had an AHR of 4.32 (95% CrI: 2.35–8.27) for the current values and 5.64 (95% CrI: 4.24–10.82) for the quarterly rate of change. Additionally, age ≥ 65 years (AHR = 4.62; 95% CrI: 1.83–12.21), HDL-C < 40 mg/dL (AHR = 3.32; 95% CrI: 1.73–7.86), diabetes mellitus (AHR = 3.08; 95% CrI: 2.01–9.54), and proteinuria positivity (AHR = 2.85; 95% CrI: 1.48–5.55) were significant predictors of the incidence of CKD. These findings highlight the importance of close BP monitoring in Ethiopian hypertension clinics.

**Conclusion:**

The incidence of CKD in this study was relatively high compared with that reported in previous similar studies conducted in Ethiopia. Our findings confirm that time-dependent systolic BP and diastolic BP trajectories are strongly associated with an increased risk of CKD. Additionally, age, low HDL-C levels (<40 mg/dl), the presence of diabetes mellitus, and proteinuria were identified as significant predictors of CKD. Therefore, effective CKD prevention among hypertensive patients in Ethiopia hinges on regularly checking both their current blood pressure levels and how those levels change over time. We also need to keep a close eye on older patients (65 + years), low HDL‐C, diabetes, and proteinuria to catch those at highest risk early and step in with care.

## Introduction

HTN is defined as a condition in which systolic blood pressure (SBP) measures ≥ 140 mmHg and/or diastolic blood pressure (DBP) measures ≥ 90 mmHg across 2–3 office visits spaced 1–4 weeks apart [1]. It is a major global public health challenge [2], affecting approximately 1.28 billion people worldwide in 2023 [3]. In Africa, particularly Sub-Saharan Africa (SSA), an estimated 74.5 million adults live with HTN [4]. In Ethiopia, the pooled prevalence of HTN was 19.6% in 2022 [5]. Poorly managed HTN can lead to various micro- or macrovascular complications, including coronary artery disease, peripheral arterial diseases, stroke, nephropathy, and CKD [6].

CKD is defined as kidney damage and/or an estimated glomerular filtration rate (eGFR) of less than 60 mL/min per 1.73 m^2^, persisting for ≥ 3 months [7]. Globally, the pooled prevalence of CKD among HTN patients is 21% [8], whereas in Africa, 33.6% of hypertensive patients are affected by CKD [9]. In Ethiopia, the prevalence of CKD among HTN patients is 21.7% [10]. CKD imposes a significant economic burden due to treatment costs [11]. It is also linked to impaired quality of life, reduced life expectancy across all age groups, increased premature deaths from cardiovascular disease, and progressive loss of kidney function, which may culminate in kidney failure or end-stage renal disease (ESRD) [12,13].

Risk factors for CKD among hypertensive patients include age ≥ 65 years [14,15], sex [16], uncontrolled HTN [15,17], level of proteinuria [17], and the presence of diabetes mellitus (DM) [15,18].. BP is a crucial clinical biomarker for diagnosing and managing hypertension and plays a key role in monitoring disease progression and preventing complications such as CKD [19]. Studies have also revealed that BP levels are strongly correlated with CKD, and optimal BP control can reduce the risk of renal impairments [20]. However, screening for CKD is not performed as recommended for hypertensive patients in resource–limited areas owing to challenges with accessibility and affordability.

Therefore, monitoring longitudinal markers is essential for tracking metabolic abnormalities and poorly controlled BP and provides meaningful prognostic information that can help to classify patients on the basis of their risk of complications and guide adjustments to treatment strategies.

Previous studies conducted in Ethiopia relied on separate analyses that ignored the dependency and association between longitudinal BP and the incidence of CKD. A joint modeling approach is preferred over separate survival models [21], as it provides more efficient estimates of treatment effects on CKD risk while accounting for longitudinal BP markers measured with error. Compared with the frequentist approach, the Bayesian approach offers more intuitive and meaningful inferences, better addresses complex research questions, and is better suited for clinical decision-making. Hence, this study employs a Bayesian joint modeling framework to evaluate the interdependence between changes in BP and CKD risk.

## Methods and materials

### Study design, settings and period

An institution-based retrospective follow-up study was conducted from May 1, 2014, to May 1, 2023, among HTN patients at the University of Gondar Comprehensive Specialized Referral Hospital (UGCSRH). UGCSRF serves more than five million residents in North Gondar Zone and neighboring zones. Annually, approximately 32,786 patients are under chronic follow-up at the hospital, including 4,065 HTN patients. The data for this study were collected between June 1 and July 1, 2024.

### Data sources

The study relied solely on secondary data. Sociodemographic characteristics, along with baseline clinical data, treatment records, and laboratory findings, were extracted from patient medical records.

### Study population

All newly diagnosed HTN patients at UGCSRH from May 2014 to May 2023 were included in the study. However, those whose date of initiation was not recorded, those who had only one BP measurement, and newly diagnosed patients with CKD at the start of follow-up were excluded.

### Sample size determination and sampling procedure

The sample size was determined for two outcomes: longitudinal and survival

#### Sample size for longitudinal outcomes.

The sample size was determined by considering the following statistical assumptions: two-sided significance level (α = 5%), power of 80%, number of repeated measurements (J = 15), within-subject correlation (ρ = 0.5), largest Cohen’s effect size (δ = 0.8), variance in SBP ($\sigma_{\text{1}}^{\text{2}}$ = 15.0348), and variance in DBP ($\sigma_{\text{2}}^{\text{2}}$ = 9.5268). The sample size was calculated via the following formula [22]:


$$n=\frac{{4\left(z_{\frac{\alpha }{2}}+z_{\beta }\right)}^{2}\times \sigma^{2}\left(1+\left(J-1\right)\times \rho \right)}{J\delta^{2}}$$

The variance of SBP and DBP ($\sigma_{\text{1}}^{\text{2}}$ and $\sigma_{\text{2}}^{\text{2}}$) and the number of repeated measurements (J) were obtained from a study conducted at the Felege Hiwot Referral Hospital [23], Ethiopia. On the basis of these parameters, the calculated sample sizes were 393 for SBP and 249 for DBP.

#### Sample size for survival outcome.

Sample size determination for the survival outcome in this study was based on the following formula [24]:


$$n=\frac{4{\left(Z_{a/2}+Z_{1-\beta }\right)}^{2}}{P\theta_{R}^{2}}$$

where ***n*** is the required number of patients included in the study, α is the level of significance, set at 5%, 1 − β is the power of the test, set at 80%, P is the probability of patients expected to develop CKD, with a value of 0.176, and $\theta_{R}=\text{ln}(\text{HR}$ is the log of the hazard ratio (HR), with a value of 0.69 on the basis of [25,26]. The calculated sample size was 373.

Finally, the largest sample size (393) was selected. Adding 10% contingency, the final sample size required for this study was 433.

A sampling frame was prepared by collecting the medical record numbers (MRNs) of hypertensive patients from the hospital’s registration book. The study participants were then selected via a computer-generated simple random sampling method.

### Study variables

The dependent variables for the longitudinal submodels were SBP and DBP, measured in millimeters of mercury (mmHg). These variables were recorded from the start of treatment (baseline) and repeatedly measured every three months, whereas the dependent variable for the survival submodel was the time from the date of hypertension diagnosis until the occurrence of CKD, measured in months. The outcome was coded as censored (0) or event (1).

**Sociodemographic variables:** baseline age, sex, and sex. **Clinical and treatment variables** included blood glucose levels, lipid profiles, blood urea nitrogen levels, fasting blood sugar levels, serum creatinine levels, family history of hypertension, stage of hypertension, and treatment type. **Comorbidities:** Diabetic disease, acute kidney injury, coronary heart disease, and vascular diseases were considered independent variables.

### Operational definitions

**CKD** is defined as an abnormality of kidney structure or function, marked by a glomerular filtration rate (GFR)< 60 mL/min/1.73 m^2^, and is present for ≥3 months [7]. **Time to CKD:** The time between the date of hypertension diagnosis and the date of CKD development, measured in months. **Censored data** included loss to follow-up, death, transfer out, and being event-free at the end of the study. **Event:** CKD. **Loss to follow-up** was defined as the absence of patients from the follow-up for one year or more successive years from the date they last to visit the chronic illness clinic [27]**. Hypertension:** Defined as those who have a documented diagnosis of hypertension (i.e., BP ≥ 140/90 mmHg) or are on antihypertensive medications. The American Heart Association (AHA) categorizes hypertension into four stages [28].

**DM:** If the DM is recorded in the patient’s medical records by the physicians. **Coronary heart disease (CHD):** Patients were considered to have CHD if they had a documented history of a coronary event, coronary revascularization procedure, or a diagnosis established by a cardiologist. **Vascular disease (VD):** Patients were considered to have VDs if they had a documented history of cerebrovascular accident or transient ischemic attack or a documented history of peripheral arterial disease or revascularization for peripheral vascular disease. **All lipid profiles***—high*-density lipoprotein cholesterol (HDL-C), low-density lipoprotein cholesterol (LDL-C), triglyceride, and total cholesterol—were categorized for analysis on the basis of the guidelines from the Mayo Clinic and World Health Organization (WHO) [28]. **Proteinuria:** Urine dipstick results were used to determine urine albumin levels, which were categorized as negative, + 1, + 2, + 3, or +4 [29].

### Data collection and quality control

A structured English-language data collection checklist was developed on the basis of existing medical records of hypertensive patients and previous similar studies. To ensure accurate data extraction, four nurses with experience in outpatient chronic care clinics as data collectors and two health officers as supervisors were involved in the data collection process. To maintain data quality, the data extraction tool was pretested for consistency and completeness using 5% of patient charts one week before the actual data collection period. Necessary adjustments were made for the final data collection sheet by excluding variables not documented in the charts, such as educational status and smoking history. A two-day training session was conducted for the data collectors prior to data collection. The training covered the study objectives, data extraction procedures, and use of the data extraction form. Each component of the tool was explained clearly to the data collectors. Throughout the data collection period, the data extraction process was closely monitored by supervisors and the principal investigator. After each data extraction form was completed, the completeness of the data was checked, and corrections were made before the patient charts were returned.

### Data management and analysis

Continuous and nonnormally distributed variables are summarized as medians and interquartile ranges (IQRs). Categorical variables are presented as frequencies and percentages. The survival experience of the patients was assessed via the log rank test and Kaplan–Meier survival curves. A Cox PH model was fitted to identify the risk factors. The proportional hazard (PH) assumption was assessed via Schoenfeld residuals before fitting the survival submodel. Variables with a p value of ≤ 0.25 in the bivariable Cox regression analysis were included in the final survival submodel. The goodness of fit of the model was assessed via the Martingale residual technique.

Exploratory analysis was conducted to visualize the patterns of individual profiles and average evolution changes graphically. The normality of the data was assessed via Q‒Q plots. For each longitudinal outcome (SBP and DBP), a linear mixed effect model with a random intercept only and both a random intercept and slope was fitted. The best-fitting model that predicts the mean change in SBP and DBP measurements over time was selected via the Akaike information criterion (AIC) and Bayesian information criterion (BIC). Consequently, the random intercept and slope model was chosen because it appropriately predicted the average change in SBP and DBP measurements over time.

The association parameters (alpha values) from the final fitted joint model were used to evaluate the relationships between longitudinal biomarkers and the risk of CKD. The joint models were fitted via different parameterizations to accurately capture the relationships between changes in SBP and DBP over time and their associations with the risk of CKD via the Bayesian approach. The optimal parameterization for the data was selected via the deviance information criterion (DIC) and the widely applicable information criterion (WAIC). A 95% credible interval was employed to determine statistically significant predictors.

The data were entered into the Kobo data collection tool, and all analyses were performed via R version 4.3.1, which uses the *nlme* [30], *survival* [31], and *JMBayes2* packages [32,33].

### Bayesian joint model specification

#### Longitudinal submodel.

Longitudinal data arise when a single outcome or a set of different outcomes on the same unit is measured repeatedly over time [34]. Linear mixed models (LMMs) are widely used in the literature for modeling a longitudinal Gaussian outcome and provide a flexible modeling framework based on a random effects approach [35]. In this study, before joint modeling, an LMM was employed to develop an appropriate longitudinal submodel for repeatedly measuring BP. The goal was to identify covariates that significantly influenced the mean changes in BP measurements over time. To measure the effect of the longitudinal covariates on the risk of an event, it is necessary to estimate the true unobserved values of the longitudinal covariates,$\eta_{ki}(t),$ and reconstruct the complete longitudinal history, $M_{ki}(t),,$ for each subject. This is achieved by postulating a suitable mixed-effects model to describe the subject-specific time evolution, as represented by the following notation [22].


$$\left\{ \;\;\;\;\;\begin{array}{c} \;\;\;\;\;\;\;\;\;\;\;\;\;\;\;\;\;\;\;\;\;\;\;\;y_{ki}(t)=\eta_{ki}(t)+\epsilon_{ki}(t),\\ {\;\;\;\;\;\;\;\;\;\;\;\eta }_{ki}(t)=x_{ki}^{⊤}(t)\beta_{k}+z_{ki}^{⊤}(t)b_{ki},\\ b_{ki}\sim N(\text{0},D),\epsilon_{ki}(t)\sim N\left(\text{0},\sigma_{\varepsilon }^{\text{2}}\right) \end{array}\right.$$ (1)

where $y_{ki}$ is the $k^{th}$ observed longitudinal response for the $i^{th}$ subject, $X_{ki}(t)$ is the design matrix for the fixed effects $\beta_{k}$, $Z_{ki}(t)$ is the design matrix for the random effects$b_{ki}$, and D is the variance‒covariance matrix of the random effects. The error terms,$\varepsilon_{ki}(t),$ are assumed to be mutually independent, independent of the random effects, $b_{ki},$ and normally distributed with a mean of zero and variance of $\sigma_{\varepsilon }^{\text{2}}$.

#### Survival submodel.

The hazard function of the survival model is used to explain the probability that the event has occurred by timet. A common approach for modeling time‒event data in a joint model is the Cox proportional hazards model [36]. The Cox proportional hazard model expresses that the hazard of an event at time t is given by [36]:


$$h_{i}(t)=h_{\text{0}}(t)\text{exp}\left(W^{\text{T}}\Upsilon \right)$$ (2)

where $h_{\text{0}}$ is the baseline hazard function, W is the matrix of baseline covariates, and the term γ is the corresponding vector of regression coefficients.

#### Joint model formulation.

The foundation of the joint modeling framework is the assumption that both the longitudinal and time-to-event processes are driven by unobserved and shared random effects. Conditional on these random effects, the two processes are assumed to be independent [37]. The intuition behind joint models is straightforward: rather than using the actual observed repeated measurements directly as time-dependent covariates in a relative risk model, the true underlying longitudinal trajectory is first estimated via a mixed-effects model. This estimated trajectory (or its functions) is then incorporated into the relative risk model [38].

A joint model of longitudinal and time-to-event data can effectively evaluate the impact of the longitudinal covariate, measured with error, on the time to an event of interest. In this study, we assessed the ability of a longitudinal biomarker, BP, to predict the risk of CKD. The joint model is specified formally as follows [36]:


$$\;\;\;\;\;\left\{ \begin{array}{c} {\;\;\;\;\;\;\;\;\;\;\;\;\;\;\;\;\;\;\;\;\;\;\;\;\;\;\;\;\;\;\;\;\;\;\;\;\;\;\;\;\;\;\;\;\;\;\;\;\;\;\;\;\;\;\;\;\;\;\;\;\;\;\;\;\;\;\;\;\;\;\;\;\;\;\;\;\;\;\;\;\;\;\;\;\;\;\;\;\;\;\;\;\;\;\;\;\;\eta }_{ki}(t)=x_{ki}^{⊤}(t)\beta_{k}+z_{ki}^{⊤}(t)b_{ki}\\ h_{i}\left(t|M_{i}(t),w_{i}\right)=h_{\text{0}}(t)\text{exp}[\gamma^{⊤}w_{i}+\sum_{k=\text{1}}^{K}\sum_{l\text{1}}^{L_{k}}f_{kl}\left(\alpha_{kl},w_{i},b_{ki},M_{i}(t)\right)]\\ \;\;\;\;\;\;\;\;\;\;\;\;\;\;\;\;\;\;\;\;\;\;\;\;\;\;\;\;\;\;\;\;\;\;\;\;\;\;\;\;\;\;\;\;\;\;\;\;\;\;\;\;\;\;\;\;\;\;\;\;\;\;\;\;\;\;\;\;\;\;\;\;\;\;\;\;\;\;\;\;\;\;\;\;\;\;\;\;\;\;\;\;\;\;\;\;\;\;\;\;\;\;\;\;\;\;\;\;\;\;b_{ki}=(\begin{array}{c} b_{\text{1}i}\\ b_{\text{2}i} \end{array})\sim N\left(\text{0},D\right) \end{array}\right.$$ (3)

where $M_{i}(t)=\left\{M_{\text{1}i}(t),\ldots ,M_{ki}(t)\right\}$ represents the history of the true unobserved longitudinal process up to time t$M_{ki}(t)=\{\eta_{ki}(s)|\text{0}\leq s<t\}$ denotes the longitudinal trajectory for the $k^{th}$ outcome, and $w_{i}$ contains baseline covariates with corresponding regression coefficients γ. Similarly, the parameter $\alpha_{k}$ quantifies the effect of the underlying longitudinal outcome on the risk for an event. In this study, *k = 1* and *2* correspond to SBP and DBP, respectively.

### Association structures

There are four common association structures (parameterizations) in the joint modeling approach: current value, slope, current value and slope, and area [21,36,39].

### Bayesian multivariate joint model

The Bayesian joint model relies on the full posterior distribution for statistical inference [40]. The posterior distribution is constructed by combining the likelihood and prior distributions, with vague or flat priors used in this study to minimize their influence relative to the observed data. Owing to the absence of prior information on the parameters of interest, noninformative priors are assigned. Noninformative priors for all the parameters were chosen. We used the default prior distributions except for the variance–covariance matrices *(****D****).* Specifically, a normal distribution with a mean of 0 and variance of 0.001 is used for the fixed effects of the longitudinal submodel ${(\beta }_{k}$), regression coefficients of the survival submodel (γ), and the association parameters $(\alpha_{k})$. We used inverse gamma prior to shape 1 and scale 0.005 for the precision parameters of the survival and longitudinal submodels, independent gamma priors were assumed for the parameters of the baseline risk function, and an inverse Wishart distribution was used for the variance‒covariance matrices (**D**).

Convergence was assessed via trace plots, the effective sample size (ESS), and Gelman–Rubin statistics, as recommended by [41,42].

### Ethical consideration

Ethical approval was obtained from the Institutional Review Committee of the University of Gondar with reference number/IPH/2880/08/2024. Before we accessed the data from medical records, they were fully anonymized. Owing to the retrospective nature of the study, the Institutional Review Committee of the Institute of Public Health, University of Gondar waived the need to obtain informed consent. In addition, official permission was obtained from the UGCSRH clinical director. The privacy of the patients’ medical records was maintained, names were not included, and the checklist was kept locked.

## Results

### Baseline characteristics of the study participants

A total of 408 participants were included in the study. Twenty-five records with incomplete information, such as unknown date of enrollment and outcome of interest, and those with CKD at the time of HTN diagnosis, were excluded. Among them, 223 (54.66%) were male, and 259 (63.48%) were urban residents. The median age of the participants was 57 (IQR = 17) years. A quarter of the respondents, 98 (24.02%), had a family history of HTN. The majority of the newly diagnosed HTN patients, 255 (62.50%), had stage-two hypertension at the baseline. Nearly half of the HTN patients, 187 (45.83%), started treatment with a combination of more than two antihypertensive regimens. Acute kidney injury and positive proteinuria were found in 109 (26.72%) and 128 (31.37%) of the study participants, respectively. Approximately 127 (32.23%) participants had baseline fasting blood sugar (FBS) levels ≥ 125 mg/dl, and 208 (52.79%) participants had baseline blood urea nitrogen (BUN) levels (≥24 mg/dl). Approximately 144 (37.31%) and 177 (45.85%) of the patients had elevated total cholesterol and triglyceride levels, respectively. More than half of the patients, 249 (66.94%), had an LDL-C level ≥ 100 mg/dl, whereas 122 (32.80%) had an HDL-C level <40 mg/dl. The median (IQR) serum creatinine level was 0.92 (0.7, 1.16) mg/dl. In terms of comorbidities, 122 (29.91%), 123 (30.15%) and 109 (26.72%) of the participants had diabetes mellitus (DM), coronary heart disease (CHD), and a history of vascular disease (VD), respectively (Table 1).

**Table 1:** Sociodemographic and clinical characteristics of HTN patients at UGCSRH, 2014–2024.

Variable	Categories	CKD status		Total (%)
		Censored (%)	Event (%)	
Sex	Female	198(48.53)	25(6.13)	223(54.66)
	Male	152(37.25)	32(8.09)	185(45.34)
Residence	Rural	127(31.13)	22(5.39)	149(36.52)
	Urban	223(54.66)	36(8.82)	259(63.48)
FBS^**a**^	<125	230(58.38)	37(9.39)	267(67.77)
	≥125	106(26.90)	21(5.33)	127(32.23)
BUN^**a**^	<24	177(44.91)	9(2.28)	186(47.21)
	≥24	159(40.36)	49(12.44)	208(52.79)
Total Triglyceride^**b**^	<150	191(49.48)	18(4.66)	209(54.15)
	≥150	137(35.49)	40(10.36)	177(45.85)
Total Cholesterol^**b**^	<200	213(55.18)	29(7.51)	242(62.69)
	≥200	115(29.8)	29(7.51)	144(37.31)
HDL_C^**c**^	≥40	228(61.29)	22(5.91)	250(67.20)
	<40	86(23.12)	36(9.68)	122(32.80)
LDL_C^**c**^	<100	110(29.57)	13(3.49)	123(33.06)
	≥100	204(54.840	45(12.10)	249(66.94)
Proteinuria	Negative	270(66.18)	10(2.45)	280(68.63)
	Positive	80(19.19)	48(11.76)	128(31.37)
Acute kidney injury	No	282(69.12)	17(4.17)	299(73.28)
	Yes	68(16.67)	41(10.05)	109(26.72)
Diabetes Mellitus	No	276(67.65)	10(2.45)	286(70.09)
	Yes	74(18.14)	48(11.76)	122(29.91)
History of CHDs	No	259(63.48)	26(6.37)	285(69.85)
	Yes	91(22.30)	32(7.84)	123(30.15)
History of VDs	No	263(64.46)	36(8.82)	299(73.28)
	Yes	87(21.32)	22(5.39)	109(26.72)
Stage of HTN	Stage 1	48(11.76)	5(1.23	53(13.48)
	Stage 2	226(55.39)	29(7.11)	255(62.50)
	Hypertensive Crisis	76(18.63)	24(5.88)	100(24.51)
FHHTN	No	261(63.97)	49(12.01)	310(75.98)
	Yes	89(21.81)	9(2.21)	98(24.02)
Treatment combinations	Single Drug	113(27.70)	25(6.13)	138(33.83)
	≥Two Drugs	237(58.67	33(8.09)	270(66.17)
Age	Median (IQR)	57(48.25,64.75)	57.5(54.25,64.75)	57(48,65)
Creatinine	Median (IQR)	0.89(0.7,1.08)	1.4(1.03,1.9)	0.92(0.7,1.16)

mg/dl: milligrams per deciliter, ^a^: (n = 394), ^b^: (n = 386), ^c^: (n = 372), HDL_C = high-density lipid cholesterol, LDL_C = low-density lipid cholesterol.

### Exploring blood pressure changes

**Fig 1:**
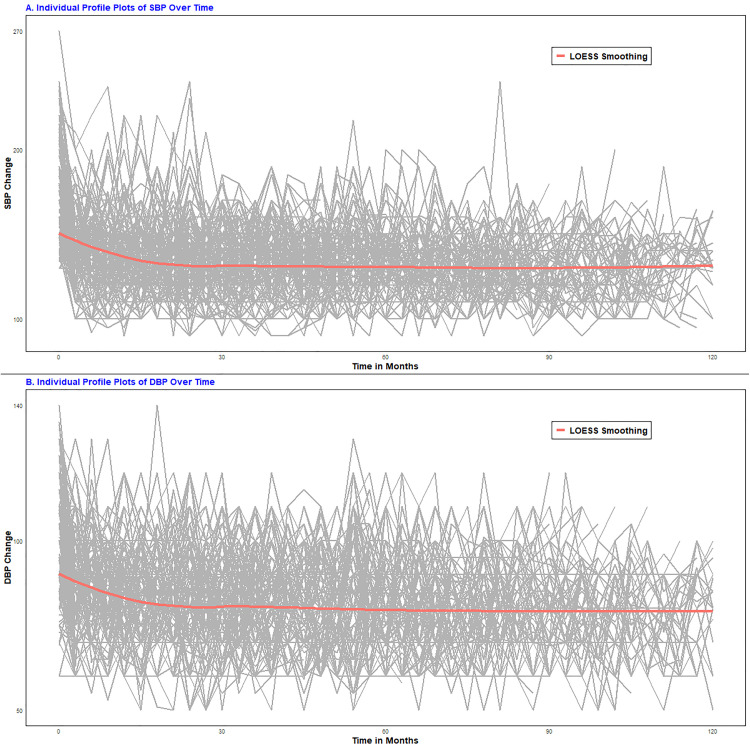
Individual profile plots for both longitudinal outcomes, SBP (Fig. 1A) and DBP (Fig. 1B), among HTN patients at UGCSRH, 2014–2024.

To explore the associations between the SBP and DBP measurements over time, individual profile plots were employed. The locally estimated scatterplot smoothing (LOESS) smoothing technique was applied to those plots to examine the mean changes in SBP and DBP measurements over time (Fig 1).

**Fig 2:**
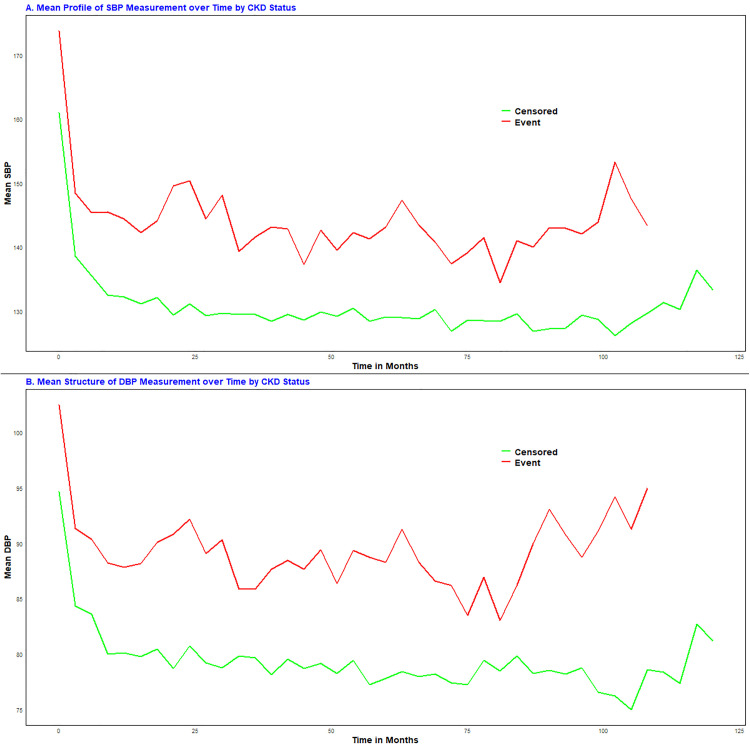
Mean profile plots of SBP and DBP by CKD status for HTN patients at UGCSRH, Northwest Ethiopia, 2014–2024.

The plots reveal significant within- and between-subject variations in BP measurements over time. Individual trajectories of BP for patients indicate considerable differences in both SBP and DBP at baseline and throughout the follow-up period, suggesting the need for a mixed-effects model that incorporates both random intercepts and slopes. The red line, representing the mean structure of BP measurements over time via the LOESS technique, indicates a linear trend in the mean SBP and DBP measurements over time. Furthermore, as shown in Fig 2A and Fig 2B, patients with higher systolic and diastolic BPs tended to have a greater risk of chronic kidney disease (CKD) over time. The red and green lines represent the mean blood pressure profiles for the CKD (event) and censored groups, respectively (Fig 2).

### Sensitivity analysis for missing data

When blood pressure measurements were not observed during a 3-month period, these values were considered missing. In this study 36 (9%) participants had missing BP measurements and thus were dropped in the complete‐case analysis. A key challenge in handling missing data in longitudinal studies is that the observed data alone cannot distinguish between a missing at random (MAR) and a missing not at random (MNAR) dropout mechanism. To address this issue, we conducted a sensitivity analysis to examine the impact of different missing data handling methods on our results. Specifically, we compared parameter estimates and standard error estimates to assess the robustness of our findings. The results showed that the parameter estimates from the complete case analysis were not significantly different from those obtained via multiple imputations. Consequently, we chose to proceed with the complete case analysis approach within the Bayesian joint model framework, utilizing the current value parameterization, as outlined in (Table 2).

**Table 2:** Comparison of the Bayesian multivariate joint model with the current value and slope parameterization under multiple imputation and complete case analysis missing handling methods.

Methods	Parameterizations	WAIC	DIC
Complete case analysis	Current value and slope	33, 566.57	32,084.22
Multiple imputation	Current value and slope	93,732.63	89,759.43,

WAIC and DIC agree that the Bayesian joint model with the current value parameterization with complete case analysis missing handling approach has better predictive ability.

**Fig 3:**
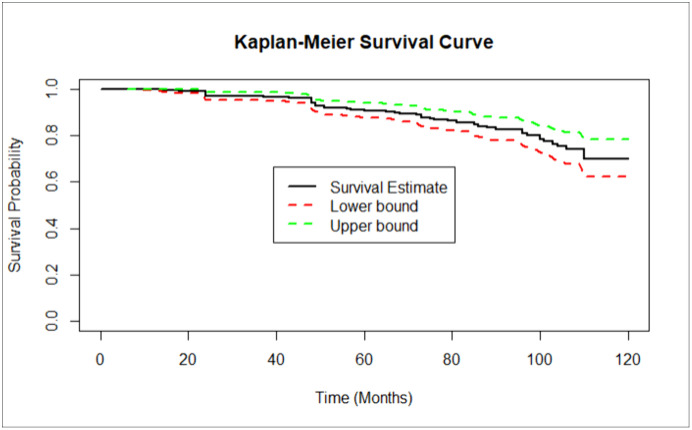
The plot of the overall estimate of Kaplan–Meier survivors for time to CKD among hypertensive patients at UGCSRH, 2014–2024. The black line indicates the survival estimate, and the red and green lines represent the lower and upper boundaries of the estimate, respectively.

**Incidence of CKD**: The patients were followed for a minimum of 6 months and a maximum of 120 months, with a median follow-up time of 70 months (IQR = 43–97). Among 408 patients, 58 (14.22%; 95% CI: 11.1–18.1) developed CKD during 2322.83 person-years (PY) of observation. The incidence density was 2.5 cases per 100 PY (95% CI: 1.89–3.14). The survival probability decreased as follow-up increased. The cumulative survival probabilities of the patients at 24, 48, 72, and 96 months were 97.1%, 93.8%, 89%, and 81%, respectively. Approximately half (51.72%) of the CKD patients developed within the first five years. The median survival time or the survival time at which the cumulative survival function was equal to 0.5 could not be determined (Fig 3).

### Model comparisons and convergence of the algorithm

The Bayesian joint model with current value and slope parameterization was selected as the best-fit model (Table 3). Convergence of the Bayesian joint model was achieved via 12 knots, three Markov chain Monte Carlo chains, a thinning interval of 12, a burn-in period of 25,000 iterations, and a total of 100,000 iterations.

**Table 3:** Comparison of Bayesian multivariate joint models for HTN patients at UGCSRH, 2014–2024.

Parameterizations	WAIC	DIC
Current value	34,406.43	33,044.7
Current value and slope	33, 566.57	32,084.22
Area	34,560.17	33,628.5
Area and current value	35,908.21	33,734.1
Slope	37,154.74	35,302.3

### Predictors of the incidence of CKD

The joint model reveals factors associated with both changes in BP and the incidence of CKD. The results revealed that the current value and the quarterly rate of changes in both SBP and DBP were significantly associated with the risk of CKD (Table 5), meaning that each one mmHg increase in the current value of SBP was associated with a 6.25-fold higher risk of developing CKD (AHR = 6.25, 95% CrI: 2.85, 9.85), whereas a one mmHg increase in the rate of the SBP trajectory increased the hazard of CKD by 3.75-fold (AHR = 3.75, 95% CrI: 3.16, 7.95) at the same time t. Similarly, a one mmHg increase in the current value of DBP was associated with a 4.32-fold greater risk of developing CKD (AHR = 4.32, 95% CrI: 3.35, 8.27), and a one-unit increase in the rate of the DBP trajectory increased the risk of CKD by 5.64-fold (AHR = 5.64, 95% CrI: 4.24, 10.82), provided that the true and observed values remained constant.

**Table 5:** Fitted Bayesian multivariate joint model of longitudinal submodels results under the current value and slope parameterizations for HTN patients in UGCSRH, 2014–2024.

Longitudinal submodels				
	SBP sub model		DBP sub model	
Fixed effects	Mean (95% CrI)	Rhat	Mean (95% CrI)	Rhat
Intercept	11.99(11.83,12.16)	1	9.19(8.88,9.46)	1
(SBP, DBP) time 1	−0.130(−4.30, −0.26)	1	−0.14(−0.21, −0.07)	1
(SBP, DBP) time 2	−1.41(−1.55, −1.26)	1	−0.95(−1.09, −0.82)	1
(SBP, DBP) time 3	−0.37(−0.49, −0.23)	1	−0.37(−0.48, −0.26)	1
**Sex**				
Female	1			
Male	0.072(0.01,0.03)	1	0.09(−0.03,0.21)	1
**History of DM**				
No	1			
Yes	0.17(0.11, 0.25)	1	0.45(0.33,0.56)	1
**History of CHD**				
No	1		1	
Yes	0.12(0.03,0.18)	1	0.38(0.25,0.51)	1
**History of AKI**				
No	1			
Yes	0.04(1.99,5.23)	1	0.07(−0.06,0.19)	1
**Stage of HTN**				
Stage 1	1		1	
Stage 2	0.16(0.07,0.26)	1	0.15(−0.01,0.32)	1
Hypertensive crisis	0.46(0.33,0.57)	1	0.5(0.3,0.71)	1
**Drug regimens**				
Single	1		1	
≥Two drugs	−0.02(−0.09,0.05)	1	−0.04(−0.15,0.08)	1
**HTN Duration**	−0.13(−0.2, −0.05)	1	−0.08(−0.19,0.04)	1
**Random effect components**				
Std. Dev. (Intercept)	0.36		0.497	
Std. Dev. (Time)1	0.54		0.37	
Std. Dev. (Time)2	0.89		0.77	
Std. Dev. (Time)3	0.53		0.45	
Std. Dev. (Residuals)	0.62		0.53	

In addition, covariates such as baseline age, HDL-C level, history of diabetes mellitus, and proteinuria were significant predictors of CKD incidence among HTN patients. Keeping other variables constant, the hazard of developing CKD for newly diagnosed HTN patients aged ≥ 65 years was 4.62 times greater than that for patients aged 18–64 years (AHR = 4.62, 95% CrI: 1.83, 12.21). Given that other variables are constant, the risk of experiencing CKD for HTN patients with HDL-C < 40 mg/dl was 3.32 times greater than that for those with HDL-C ≥ 40 mg/dl (AHR = 3.32, 95% CrI: 1.73, 7.86). After adjusting for other variables in the model, the hazard of CKD for DM patients was 3.08 times greater than that for patients who were not diabetic (AHR = 3.08, 95% CrI: 2.01, 9.54). The risk of experiencing CKD for HTN patients with positive proteinuria was found to be 2.85 times greater than that for HTN patients with negative proteinuria (AHR = 2.85, 95% CrI: 1.48–5.55), as presented in Table 4.

**Table 4:** Fitted Bayesian multivariate joint model of survival submodel results under current value and slope parameterization for HTN patients in UGCSRH, 2014–2024.

Survival submodel				
Covariates		AHR [95%CrI]	ESS	Rhat
**Age**				
18-64		1		
≥65		4.64[1.83,12.21]	66,168.12	1
**Sex**				
Female		1		
Male		0.43 [0.59,4.39]	63,428.61	1
**Triglycerides (mg/dl)**				
<150		1		
≥150		1.33 [0.59,2.98]	35,863.81	1
**Total cholesterol (mg/dl)**				
<200		1		
≥200		1.06 [0.56,1.99]	47,134.17	1
**HDL_C (mg/dl)**				
≥40		1		
<40		3.32 [1.73,7.86]	81,693.11	1
**BUN (mg/dl)**				
<24		1		
≥24		1.69 [0.64,5.05]	49,343.01	1
**History of DM**				
No		1		
Yes		3.08 [2.01,9.54]	46,341.45	1
**History of CHD**				
No				
Yes		1.89 [.98, 4.44]	71,049.95	1
**History of VDs**				
No		1		
Yes		1.16 [0.43,2.53]	82,831.62	1
**Proteinuria**				
Negative				
Positive		2.85[1.48, 5.55]	63,375.97	1
**History of AKI**				
No		1		
Yes		1.83 [0.85,4.25]	84,218.97	1
**Treatment combinations**				
Single regimen		1		
≥Two regimens		0.78 [0.29,1.91]	61,356.04	1
**Association parameters**				
**Time varying covariates**	**Parameterizatio**n			
𝐒𝐁𝐏	Current value	6.25 [2.85, 9.83]	73,719.75.	1
	Slope	3.75[3.16, 7.95]	67,919.67.	1
𝐃𝐁𝐏	Current value	4.32 [2.35, 8.27]	83,437.32	1
	Slope	5.64[4.42, 10.82]	72,735.83	1

**Note**: **Rhat** = potential scale reduction factor, ESS = effective sample size, CrI = credible interval

Since our main aim was survival components, the longitudinal submodels are summarized in (Table 5).

## Discussion

CKD is a major public health concern and a leading cause of renal impairment, hospitalization, morbidity, and mortality. This study investigated the incidence and predictors of CKD, as well as the effect of longitudinal BP changes on the risk of CKD, using a Bayesian joint modeling approach.

Nearly half of the CKD patients developed within the first five years. This may be attributed to the fact that most HTN patients only seek medical care after experiencing complications, such as renal impairment (RI), as the disease often remains asymptomatic until it has advanced stages [43]. Therefore, it is better to screen for CKD at the time of HTN diagnosis since most HTN patients develop CKD during the early period of follow-up.

In this study, the proportion of CKD cases was 14.22%, with an incidence rate of approximately 3 cases per 100 PYs of observation. This study revealed a greater incidence of CKD than studies conducted in Korea (1.21 per 100 PY) [44], the Netherlands (1.22 per 100 PY) [45], and Saudi Arabia (1.65 per 100 PY) [25]. However, the incidence rate in this study was lower than that reported in studies performed in Japan (5.41 per 1,00 PY) [46]. This discrepancy could be explained by differences in the study period, diagnostic methods used in the studies, and denominator population [12].

Our findings confirmed that HTN patients aged ≥ 65 years and older face a nearly fivefold greater risk of developing CKD than those aged 18–64 years. This finding aligns with studies conducted in Malaysia [14], Algeria [47], Bangladesh [48] and systematic reviews and meta-analyses in SSA [49]. This elevated risk may be attributed to the progressive decline in the GFR after the age of 30 years [50], which decreases at an average rate of 8 mL/min/1.73 m^2^ per decade [50]. Age-related structural and functional changes in the kidneys, such as nephron loss, reduced renal blood flow, and diminished renal reserve, likely exacerbate this decline [50]. Additionally, older age is strongly associated with CKD risk factors, including cardiovascular diseases and obesity [51]. As a result, implementing CKD screening for older individuals is crucial for enabling timely and appropriate clinical interventions.

HTN patients with HDL-C levels < 40 mg/dl had a threefold greater risk of developing CKD than did those with HDL-C levels ≥ 40 mg/dl. This result is consistent with studies conducted in Sudan [52], the United States of America [53,54], and Japan [55]. This association may stem from the biological role of HDL-C in reducing atherosclerosis by transporting lipids into the liver for excretion [56], thereby preventing lipid accumulation in arterial walls. HDL-C also protects the vascular endothelium from oxidative damage, mitigating HTN-related vascular complications, including CKD [57]. Low HDL-C levels may impair these protective mechanisms, increasing susceptibility to CKD.

According to our findings, diabetic patients face a much greater risk of developing CKD than their non-diabetic counterparts do. This finding is in line with those of previous studies [14,18,58–62], including a recent study of 6,251 U.S. adults with diabetes, which emphasized the direct relationship between diabetes and CKD [43]. This increased risk is likely due to the well-established condition known as diabetic nephropathy [63]. Given this evidence, prioritizing diabetes prevention remains crucial for local healthcare providers.

In this study, the hazard of CKD for hypertensive patients with positive proteinuria was threefold greater than that for those with negative proteinuria. This finding is consistent with studies conducted in Japan [18,64], Bangladesh [48], the USA [65] and with a systematic review and meta-analysis in SSA [49]. This may result from elevated urinary protein levels due to renal damage to the glomerular capillary wall or reduced tabular protein reabsorption, leading to injury to renal tabular cells, fibrosis and interstitial inflammation, further exacerbating kidney damage [66,67]. Therefore, we strongly recommend that healthcare providers intensify their efforts to prevent and/or reverse proteinuria in their patients to mitigate the risk of CKD progression.

In this study, we found that both the current value and quarterly rate of change in BP were associated with the risk of CKD. For patients who have the same covariates in the model at the baseline and who have the same underlying values of both SBP and DBP at timet, a one mmHg increases in the current value of both SBP and DBP increases the risk of CKD by six- and fourfold, respectively, and a one mmHg increase in the quarterly rate of change in both SBP and DBP increases the hazard of CKD by approximately four- and sixfold, respectively.

As far as we know, this is the first paper using a Bayesian joint model on longitudinal BP and CKD incidence in Ethiopia, although several earlier studies on hypertensive patients have consistently demonstrated a significant association between BP levels and CKD [11,18,68–72].

This is because high BP damages small blood vessels in the kidneys [7]. This damage sets off a cycle of ischemia and oxidative stress that worsens renal function [73,74], which can ultimately lead to kidney failure [18].

The purpose of the findings of this study was to provide information for health professionals and patients, as high BP levels can be used as a signal to screen HTN patients for CKD and early detection of CKD and its complications. In addition, this study provides evidence of the factors associated with the risk of CKD. Therefore, it helps to minimize risk and maximize efforts to prevent problems. The public health importance of this study is to prevent disability, economic loss, and loss of productivity associated with CKD by identifying the variables significantly associated with CKD.

One of the strengths of this study was that it estimated the incidence of CKD and identified its predictors via advanced modeling techniques.

The main limitation of this study was that its retrospective nature restricted the inclusion of all potential predictors of CKD incidence, such as certain sociodemographic and behavioral factors, which may lead to an underestimation of effects and individual variations in CKD development. Additionally, the study assumed that all the CKD cases were caused by HTN, potentially overestimating the CKD rate.

## Conclusion

In this study, the incidence of CKD was relatively high compared with that reported in previous similar studies in Ethiopia and was influenced by multiple factors. Longitudinal blood pressure changes were significantly associated with an increased risk of CKD. In addition, age, HDL-C level, diabetes mellitus and proteinuria were significant predictors of the incidence of CKD. Therefore, Effective CKD prevention among hypertensive patients in Ethiopia hinges on regularly checking both their current blood pressure levels and how those levels change over time. We also need to keep a close eye on older patients (65 + years), low HDL‐C, diabetes, and proteinuria to catch those at highest risk early and step in with care.

### Recommendations

On the basis of the findings of the current study, the following recommendations are made for the bodies of interest.

- Health professionals should pay greater attention to HTN patients with identified risk factors for CKD.
- We also highly advise that it is better to screen for CKD at the time of HTN diagnosis since most HTN patients develop CKD during the early period of follow-up.
- Further studies on this topic, including behavioral factors such as alcohol consumption and history of primary data, with prospective studies are recommended.

## Abbreviations

AHR: Adjusted hazard ratio
AIC: Akaike’s information criterion
BIC: Bayesian information criterion
BP: blood pressure
CHD: coronary heart disease
CKD: chronic kidney disease
DBP: diastolic blood pressure
DIC: deviance information criterion
DM: diabetes mellitus
eGFR: estimated glomerular filtration rate
ESRD: end-stage renal disease
FBS: fasting blood sugar
HDL-C: high-density lipoprotein cholesterol
HTN: hypertension
IQR: interquartile range
KM: Kaplan‒Meier
LDL-C: low-density lipoprotein cholesterol
MCMC: Markov chain Monte Carlo
NCD: noncommunicable disease
SBP: systolic blood pressure
SHTN: stage of hypertension
UGCSRH: University of Gondar Comprehensive Specialized Referral Hospital
